# The Germ Theory

**Published:** 1883-07

**Authors:** 


### EDITORIAL DEPARTMENT.

### THE GERM THEORY.

DURING the past few years the question of discovering the cause of contagious diseases and of determining the the best means for their prevention and cure has been prominently before the public mind. It has become of special interest since Tuberculosis has been declared to be of the same character. The investigation by Pasteur and Koch on the subject are familiar, and the results of their labors up to the present time are calculated to inspire hope for the future. Their clashing of opinions, we trust, will but make their ultimate conclusions the more convincing. Apropos of this subject, the following from the *Medical Times* and *Gazette* will be read with interest:

"Our readers may remember that at the late International Congress of Hygiene, at Geneva, M. Pasteur made a violent attack on Dr. Koch for having questioned the conclusions at which he had arrived on the subject of preventive inoculation, and an exciting debate was expected, when the German excused himself from replying on the ground of his imperfect command of the French language, and his opponent's total ignorance of any but his own. Dr. Koch, however, promised to publish his answer in the course of a few months, and he has now made his promise good. The different spirit in which the rival investigators approach the question is highly characteristic of their respective nationalities, and the discrepancies between their conclusions are owing to their different methods of procedure, besides which, it must be borne in mind that Pasteur is a chemist and has not enjoyed the physiological and pathological training of his opponent. Pasteur assumes that all communicable diseases are parasitic. The presence of any bacterium in the body of an animal affected with such a disease is, to his mind, sufficient to identify the micro-organism with the disease. He employs fluid media for cultivation, ignoring the objections urged against them by Koch and his school, and he looks on all animals as equally fitted for experimentation, neglecting to avail himself of the differential tests that a variety of species affords. Thus, using the saliva of a dead dog for the inoculation of rabbits, he describes a new form of rabies, having failed to find bacteria in the secretions of rabid dogs while alive; whereas there is every reason to be believe that his rabbits died of ordinary septicæmia. He fell into a like error when he announced his discovery of the bacillus of canine typhoid in the nasal mucus, not the intestinal glands, of horses after death. Even as regards splenic fever, Dr. Koch disputes Pasteur's claim to priority of discovery, since his own first publication appeared in 1876, and Pasteur's not till the following year. Koch does not, as some imagine, deny the possibility of the transformation of bacilli under altered surroundings into allied forms, nor the change of pathogenic organs into innocent ones, but he insists on far more crucial and exact evidence of such change before it can be accepted as an established scientific fact, much less a law of nature. He demands that in every case we must show the parasitic nature of the disease, that the micro-organism when found shall be cultivated in solid media until all chance of accidental admixture or access of other germs is excluded, and that the disease shall be reproduced by the inoculation of the pure bacillus with spores. That such demands can be realized is proved already in the case of erysipelas, of anthrax, of splenic fever and more recently of tubercle. The Imperial Board of Health of Germany instituted a series of experiments, under the direction of Dr. Koch, to test the validity of Pasteur's assertions respecting the immunity conferred by his inoculations with mitigated virus, and his theory as to the natural mode of infection. The results of these experiments are now for the first time made public. Pasteur, as is well known, asserts that by cultivating in meat broth at a temperature of 42° or 43° C. and freely exposed to the air, its virulence is steadily lessened. To diminish the danger, he advises two successive inoculations—the first with a very feeble virus, the second with one of greater activity. These directions were strictly followed out, the animals operated on being sheep. Injections of the *premier vaccin* produced little or no constitutional disturbance, but a considerable number of the animals operated or died after inoculation with the *deuxieme vaccin.* If the survivors had been now absolutely protected, something would have been gained, but such was not the case either against artificial or natural modes of infection, for of six sheep, which, having already received the *premier* and *deuxieme vaccin*, ought to have enjoyed immunity, one died within two days of inoculation with unmitigated virus taken from a case of spontaneous splenic fever. Pasteur has advanced a theory that when the carcasses of animals that have died from any form of anthracosis or have been slaughtered, are buried, the bacilli are apt to be brought to surface by worms along with their casts; and that the bacilli gain access to the circulation of previously healthy cattle through the abrasions of the oral mucous membrane caused by pricking or coarse fodder. This Koch disputes, urging that it is only in the spore bearing stage that the microphyte is infectious, and that then mere ingestion is sufficient, breaches of surface being quite unnecessary. Soft food containing bacilli—in fact potatoes hollowed out and stuffed with infective matter—were carefully introduced into the throats of the animals. When the matter consisted of the bacilli alone, entirely free from spores, no ill affects followed; but on the experiment being repeated with spore bearing bacilli, whether fresh or such as had been dried for over a year death invariably followed within two days. To test the protection afforded by the so-called preventive inoculation against natural infections, seven sheep which had survived or resisted inoculation, with pure unmitigated virus, and thus had been thrice 'vaccinated' were fed with sporiferous matter in potatoes; two of the seven died of splénic fever on the second day. Dr. Koch, therefore asserts that, even as regards sheep and cattle, the immunity is by no means certain, while none whatever is imparted to rabbits, guinea pigs, rats and mice, and man himself may suffer repeatedly from anthracosis. No protection against subsequent infection is afforded by inoculation with the bacilli of erysipelas or relapsing fever, even after the most prolonged cultivation. So that Pasteur has utterly failed on the scanty basis of his partial successes in the case of two diseases—the so-called fowl cholera and anthracosis—to establish any law of immunity; still less has he made any progress towards realizing his dream of extending its application to the prevention of every infectious disease to which man or beast is heir. His operations are more akin to small pox inoculation than to vaccination. They involve the same danger of keeping up the disease by infection of others without conferring equal security on the individuals subjected to it. In short, Dr. Koch maintains that, considering the proved uncertainty of such immunity as is imparted, its probable short duration, and the danger to life not only in the case of the animals operated on, but to others, and to the men in contact with them, Pasteur's preventive inoculation cannot, at any rate at present, be deemed of practical and economical value."

The theory of Pasteur that cattle can be infected by germs brought to the surface of the ground by earth worms from the buried carcasses of animals who had died from anthracosis is now accepted as a settled fact. As the worm has been convicted of spreading anthrax, the ant and fly may be regarded as agents for the dissemination of disease germs. And if this be true in the case of anthrax, may it not likewise be so as regards glanders, and other infectious diseases. Quarantine regulations and vaccination afford the means of protection from contagion by living animals; are there any to prevent the dead from becoming the centers of infection? A satisfactory answer is to be found in an article in the Quarterly Journal of Veterinary Science, in India, on sanitary science by James Mills, V. S., from which we quote:

"The proper disposal of the dead seems to me a question of paramount importance, although one evidently much neglected. By burial, however deep, certain parts of the country in time must become more or less contaminated and the atmosphere rendered impure, unhealthy and dangerous for both man and beast to live in. I would strongly recommend the cremation or reducing to ashes of the carcasses of all animals that have died from such diseases as anthrax or have been destroyed for glanders, and of all refuse or objectional matter, such as dressings from wounds or sores and stable litter. I am, perhaps, late in bringing this subject to notice, but it is to be hoped not too late to do some good, for I set no better or more effectual mode of getting rid of disease germs than by fire, and, in fact, it is already evident that this is the only true way of preventing the spread of contagion. The disease germs of anthrax (one of the most fatal animal scourges we have in India, and one whose increasing prevalence, no doubt, is due to burial instead of cremation of the dead) as Pasteur has shown, can travel long distances through the medium of earth worms and retain their vitality for a very long period. Is it not urgent, therefore, on such authority as his, for us to use proper and thorough means for the destruction of these germs, and in that way endeavor to remove the cause of the disease, for while the cause exists the effects will never cease * * * * * * *

"Cinerators ought to be erected near all hospitals, cholera camps, sick lines for animals, and bazaars for the purpose of burning everything likely to convey disease or to prove insanitary. To show what can be done with these cinerators, I have been able, during the outbreak of glanders and Farcy at St. Thomas Mount, to cremate the carcasses of eleven of the horses destroyed on account of disease. The average time occupied in reducing them to ashes was about 12 hours per horse, and with no other fuel than stable litter."

All these preventive measures are of great importance when the protection of animals are concerned; but how much greater is it when it concerns man, and we know that the infectious disease are not only transmissable from animal to animal, but also from animal to man. Actinomykosis, a new disease of animals is also capable of being so transmitted, and lately it has been found that Tuberculosis has this characteristic added to its original terrors. It behooves all, therefore, who are obliged to come in contact with infected animals to observe the greatest care, and take all possible precaution to avoid inception of the disease germs.

